# Uncovering the mechanism of *Maxing Ganshi* Decoction on asthma from a systematic perspective: A network pharmacology study

**DOI:** 10.1038/s41598-018-35791-9

**Published:** 2018-11-26

**Authors:** Wenjie Song, Shenglou Ni, Yanling Fu, Yun Wang

**Affiliations:** 1 School of Traditional Chinese Medicine, Beijing University of Chinese Medicine, Beijing, 100029 China; 2 Periodicals Publishing Center, Beijing University of Chinese Medicine, Beijing, 100029 China; 3 School of Chinese Materia Medica, Beijing University of Chinese Medicine, Beijing, 102488 China

## Abstract

*Maxing Ganshi* Decoction (MXGSD) is used widely for asthma over thousands of years, but its underlying pharmacological mechanisms remain unclear. In this study, systematic and comprehensive network pharmacology was utilized for the first time to reveal the potential pharmacological mechanisms of MXGSD on asthma. Specifically, we collected 141 bioactive components from the 600 components in MXGSD, which shared 52 targets common to asthma-related ones. In-depth network analysis of these 52 common targets indicated that asthma might be a manifestation of systemic neuro-immuno-inflammatory dysfunction in the respiratory system, and MXGSD could treat asthma through relieving airway inflammation, improving airway remodeling, and increasing drug responsiveness. After further cluster and enrichment analysis of the protein-protein interaction network of MXGSD bioactive component targets and asthma-related targets, we found that the neurotrophin signaling pathway, estrogen signaling pathway, PI3K-Akt signaling pathway, and ErbB signaling pathway might serve as the key points and principal pathways of MXGSD gene therapy for asthma from a systemic and holistic perspective, and also provides a novel idea for the development of new drugs for asthma.

## Introduction

Bronchial asthma (hereinafter referred to as asthma) is the most common chronic respiratory disease in the world^1^. It affects all age groups, especially in children, and has a high morbidity rate^2^. With increasing smoking and allergy rates, air pollution, and an aging population, the incidence of asthma is rising year-on-year^3–5^. Epidemiological surveys from different countries put the prevalence of asthma at between 1% to 21%, translating to an increase by approximately 30% in the past 20 years^6^. In 2015, the World Health Organization estimated that 334 million people (4.9% of the world’s population) suffer from asthma, and 250,000 deaths each year, remaining the top twenty causes of disability in children globally^7^. Apart from clinical manifestations such as wheezing, shortness of breath, chest tightness, and cough caused by the chronic airway inflammation in asthma^8–10^, complications such as respiratory failure, cardiovascular diseases, kidney diseases, sudden death^11–15^, are also frequently encountered, which not only endangers human health, increases economic burden, but also affects societal productivity^2,16^. Research has shown asthma to be a polygenic inherited respiratory disease closely related to gene-environment interactions and has significant individual differences in clinical manifestations and pathological changes^17^, but its complex pathogenesis has not been fully elucidated. Well-acknowledged mechanisms include chronic airway inflammation, airway hyper-responsiveness, reversible airway restriction and airway remodeling caused by airway immuno-inflammation and neuro-modulation^18^. Therefore, clinical drugs like anti-inflammatories, bronchodilators, and inhaled corticosteroids are used mainly for asthma. However, their side effects and unknown pathogens that cannot be identified in time are reasons for the limitations of these drugs in the clinic^19,20^. Thus, safe and effective individualized gene therapy may be a future trend in the treatment of asthma^21^.

Traditional Chinese Medicine (TCM) has been used widely in clinical practice for over thousands of years and has the advantages of being “simple, convenient, economic and effective”. Furthermore, treatment based on pattern identification is also in accordance with the concept of individualized gene therapy in modern precision medicine^22^. Therefore, finding a solution for asthma in the domain of TCM is necessary. Our previous studies confirmed that *Maxing Ganshi* Decoction (MXGSD) is effective in relieving clinical symptoms of asthma, improving lung function, shortening treatment course, bettering quality of life, and reducing mortality rate^23^, which has been confirmed by other studies^24–26^. MXGSD, a well-known formula from the *Treatise on Febrile and Miscellaneous Diseases (Shang Han Za Bing Lun)* by famous Chinese physician *Zhang Zhongjing*, comprises four Chinese herbal ingredients: Mahuang, Xingren, Gancao, and Shigao in a 5:5:3:6 ratio^27^. Research has found MXGSD to have therapeutic effects on asthma by reducing airway inflammation, regulating mucus secretion, controlling airway smooth muscle cell proliferation, and improving hypoxia^28–32^, but its underlying pharmacological mechanism is not fully clear. As the same neuro-immuno-inflammatory cascade reactions are common in different tissues and organs, and can interact with each other, a single organ cannot be viewed separately from the whole body. For this reason, we speculate that MXGSD is effective for asthma via the regulation of the body’s different systems and homeostasis.

TCM formulas have the characteristics of being multi-component, multi-target and multi-pathway, but currently, most studies are still confined to the traditional research method of “single-drug, single-target, and single-pathway”^33–35^. Network pharmacology is a comprehensive method that includes chemo-informatics, bio-informatics, network biology and traditional pharmacology, and provides a novel strategy to uncover the bioactive components and underlying mechanisms of TCM formulas from a systemic and holistic perspective^36^. Therefore, we first applied network pharmacology to identify bioactive components and targets of MXGSD, seek common targets for asthma, and understand the underlying mechanism of the common targets. We then used topological analysis to construct a core protein-protein interaction (PPI) network of MXGSD and asthma, classified the core PPI network based on cluster analysis and further explored the core pharmacological mechanisms of MXGSD in treating asthma.

## Materials and Methods

### Data sources

#### Components of MXGSD

Data on the four herbs in MXGSD were mainly obtained from the TCM systems pharmacology database (TCMSP, http://ibts.hkbu.edu.hk/LSP/tcmsp.php, updated on May. 31, 2014) and the TCM database@Taiwan (TCM@Taiwan, http://tcm.cmu.edu.tw, updated on Mar. 25, 2014), the two largest pharmacological data platforms for TCM. They contain all herbs, chemical components and pharmacokinetic properties (namely, absorption, distribution, metabolism and excretion or ADME) information in the *Pharmacopoeia of the People’s Republic of China* (2010 edition)^37,38^. In addition, the databases of the China National Knowledge Infrastructure, Wanfang, and Pubmed were also used to supplement any other omitted components. Finally, 600 components from MXGSD were collected, including 230, 85, 280 and 5 from Mahuang, Xingren, Gancao, and Shigao, respectively (Supplementary Table S1).

#### Asthma-related targets

Data on the asthma-related targets were obtained from five databases: (i) We obtained 174 asthma-related targets from the Online Mendelian Inheritance in Man (OMIM, http://omim.org/, updated on Jan. 3, 2018), an information platform mainly designed for human genes and genetic diseases^39^. (ii) We obtained 81 asthma-related targets from the Therapeutic Target Database (TTD, http://systemsdock.unit.osit.jp/iddp/home/index, updated on Sep. 15, 2017), which contains substantial information about the therapeutic proteins, gene targets, related diseases, pathways and corresponding drugs^40^. (iii) We obtained 367 asthma-related targets from the Genetic Association Database (GAD, https://geneticassociationdb.nih.gov/, updated on Sep. 1, 2014), which provides genes associated with complex diseases and disorders^41^. (iv) We obtained 85 asthma-related targets from the Pharmacogenomics Knowledge Implementation (PharmGkb, https://www.pharmgkb.org/, updated on Dec. 28, 2017), which mainly details relationships between actionable genes and clinical drugs^42^. (v) We obtained 35 asthma-related targets from the Drugbank database (https://www.drugbank.ca, updated on Dec. 20, 2017), which offers target genes from FDA-approved drugs^43^. After removing duplicates, 546 asthma-related targets were collected altogether (Supplementary Table S2).

### Data preprocessing

#### Screening bioactive components of MXGSD

Oral administration is the most common route for TCM herbs to take effect in the gastrointestinal tract. Therefore, we selected two ADME-related models, bioavailability (OB) and drug-likeness (DL), for the bioactive components screening of MXGSD, which plays an important role in pharmacodynamics studies. The screening criteria are: OB ≥ 20% and DL ≥ 0.18^44^. In this study, 141 bioactive components of MXGSD were included, 11 from Mahuang, 10 from Xingren, 115 from Gancao, and 5 from Shigao (Supplementary Table S3).

#### Bioactive component-target prediction for MXGSD

According to different component-target prediction principles, target prediction techniques and methods can be divided into four types: (i) ligand-based prediction (chemical similarity search and pharmacophore model); (ii) receptor-based prediction (molecular docking); (iii) machine learning prediction (the database must have a clear correspondence between the molecules and targets, and the target names must be standardized), and (iv) combined prediction^45^. Based on our previous experience and considering the limitations of experimental conditions for molecular docking and machine learning predictions, we chose ligand-based prediction method for the subsequent study^43,46^.

First, we chose three common chemical component databases of TCMSP, PubChem Project (PubChem, https://pubchem.ncbi.nlm.nih.gov/, updated on Oct. 26, 2017), and ZINC (http://zinc15.docking.org, updated on Mar. 14, 2016) as the main sources of structure information on chemical components, from which we obtained the detailed information about the bioactive components of MXGSD, including canonical smiles, molecular structures, and their “mol2” files.

Second, we chose four public databases of TCMSP, Swiss Target Prediction (http://www.swisstargetprediction.ch/), STITCH (http://stitch.embl.de/), and Pharm-Mapper (http://lilab.ecust.edu.cn/pharmmapper/index.php, updated on Nov. 27, 2017), as the main sources of component-target data, which predicts targets depending on the chemical similarities and pharmacophore models of its component. After that, we obtained the target protein names of MXGSD bioactive components.

Finally, by converting target protein names of MXGSD bioactive components into gene names with the species limited into “Homo sapiens” by UniProt Knowledgebase (UniProtKB, http://www.uniprot.org/), we established the component-target data table (Supplementary Table S4).

#### Screening common targets for MXGSD and asthma

After screening the common targets of MXGSD and asthma (Supplementary Table S5), we explored the underlying mechanisms of these common targets with gene ontology (GO) and Kyoto Encyclopedia of Genes and Genomes (KEGG) pathway analysis (Supplementary Table S6).

### PPI network construction

Proteins often work together with other molecules, such as lipids, nucleic acids, and other proteins^47^. The PPI network is a network structure that physically connects proteins through molecular docking in cells^48^, and can be visually analyzed using Bisogenet, a Cytoscape plugin. The data sources came from 6 main PPI databases, namely, Database of Interacting Proteins (DIP), Biological General Repository for Interaction Datasets (BioGRID), Human Protein Reference Database (HPRD), IntAct Molecular Interaction Database (IntAct), Molecular INTeraction Database (MINT), and Biomolecular Interaction Network Database (BIND)^49^. PPI networks of MXGSD bioactive component-targets and asthma-related targets were then constructed.

### Central network evaluation

Central network evaluation is a topological method of defining the core central network. First, the two PPI networks of MXGSD bioactive component-targets and asthma-related targets were compared, before being analyzed and evaluated by CytoNCA, a Cytoscape plugin. Each node in the area of intersection was assessed with its eight typical central attributes: betweenness centrality (BC), closeness centrality (CC), degree centrality (DC), eigenvector centrality (EC), local average connectivity-based method (LAC), network centrality (NC), subgraph centrality (SC), and information centrality (IC)^50^. However, using the Cytoscape software, only six of the eight typical center attributes of CytoNCA can be used to calculate the required data, namely BC, CC, DC, EC, LAC and NC. Therefore, we selected “DC ≥ 2 × median DC” as the primary screening criteria, before finding the shortest average distance (CC), shortest paths (BC), highest eigenvector score (EC), largest average local connectivity (LAC), and biggest aggregation coefficients (NC) as the core targets^51,52^ (Supplementary Table S7). If the initial screening criteria was to satisfy six conditions at the same time, the degree of targets found cannot be considered core targets.

### Cluster analysis

Clusters refer to highly interconnected regions distilled from different, complex objects with similar underlying properties. Cluster analysis as an important classification method clearly shows the inherent laws in a PPI network^53^. Many algorithms in the cluster analysis of PPI network in Cytoscape have been reported, but previous research confirmed that the network stability of the module generated by the Molecular Complex Detection (MCODE) algorithm is superior^54^, so we chose MCODE to perform cluster analysis for the PPI network (Supplementary Table S8).

### Enrichment analysis of GO and KEGG pathway

GO (including biological process (BP), cell component (CC), and molecular functions (MF)) and KEGG pathway enrichment analysis are the common methods used to describe the characteristics of candidate targets^55^. We utilized the Database for Annotation Visualization and Integrated Discovery (DAVID, https://david.nicifcrf.gov/, updated in Mar. 2017), an online platform for the high-throughput functional annotation bioinformatics, to perform functional annotation and enrichment analysis, and recognized GO terms with Bonferroni <0.05^34^ (Supplementary Table S9) and KEGG pathways with P-value < 0.05^35^ as significant (Supplementary Table S10).

## Results

### MXGSD component-target network

**Figure 1:**
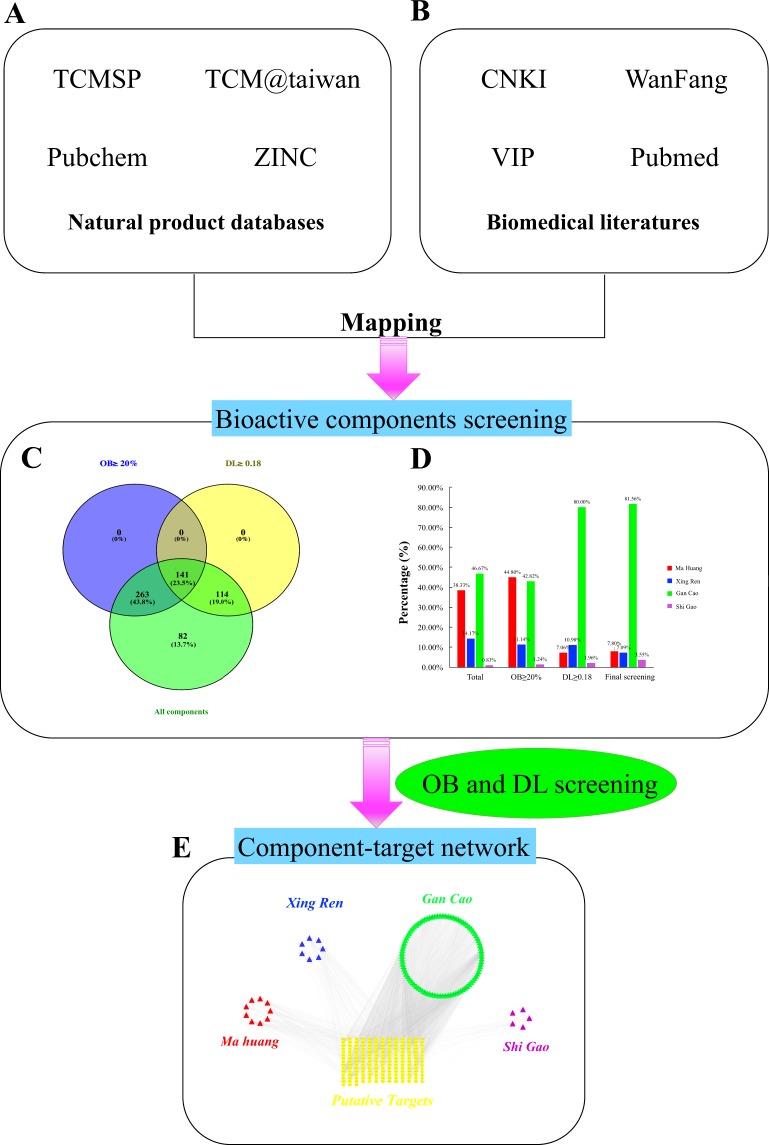
MXGSD component-targets network. Two broad categories database: (**A**) natural product databases and (**B**) biomedical literatures. (**C**) 600 constituent components (green section), and 141 bioactive components screened by two ADME-related models (blue section stands for the components of OB ≥20%, yellow section stands for DL ≥0.18). (**D**) Distributions of different herbs. (**E**) Construction of MXGSD bioactive component-putative targets visual network.

We retrieved Chinese herbal components from two broad categories of databases: natural product databases (Fig. 1A) and biomedical literature (Fig. 1B). In total, 600 components were collected, including 230, 85, 280, and 5 from the four herbs: Mahuang, Xingren, Gancao and Shigao, respectively. Among these components, O-benzoyl-L-(+)-pseudoephedrine, ()-N-methylpseudoephedrine, ()-N-methylephedrine, amygdalin, glycyrrhizin, CaSO4·nH2O (n = 0 or 2), Fe, Mn, and Zn were retrieved manually for further study owing to their reported properties^56–58^. The components were screened by two ADME-related models, OB and DL, and 141 bioactive components were included (Fig. 1C), of which 7.80% (11/141) from Mahuang, 7.09% (10/141) from Xingren, 81.56% (115/141) from Gancao, and 3.55% (5/141) from Shigao (Fig. 1D). Of these 141 bioactive components, three groups from Mahuang, Xingren, and Shigao overlapped significantly, which indicates that different herbs in a formula can share the same or similar components and targets with synergistic effects.

Generally speaking, the therapeutic and preventive effects of a compound formula rely on the synergies between the multiple components, targets and pathways^59^. Therefore, we then explored the effective targets of the 141 MXGSD bioactive components with ligand-based prediction strategy. After removing duplicates, we finally identified 186 potential targets from 118 out of 141 bioactive components. The number of these targets in Mahuang, Xingren, Gancao, and Shigao was 112, 62, 139, and 34 respectively.

To further understand MXGSD component-targets network from a systematic and holistic view, we constructed a network map using Cytoscape, version 3.2.1 (http://www.cytoscape.org)^60^ (Fig. 1E). The network contained 300 nodes and 2512 edges altogether. The degree of node represented the number of edges or targets linking with the node based on the topological analysis. In this network, we identified 56 candidate components with a median ≥21 degrees, which also proved synergistic effects in different herbs and components. Specifically, we found that quercetin, kaempferol, and apigenin acted on 172, 106 and 46 targets, respectively, and became the crucial bioactive components in MXGSD. Some studies suggested that quercetin could reduce airway inflammation and relax airway smooth muscle (ASM) by regulating some key pathways, such as mast cell signaling pathway and calcium ion pathway, which in turn manages asthma^61–63^. This may also explain the pleiotropic effects of traditional Chinese medical herbs.

### Asthma-related target network

**Figure 2:**
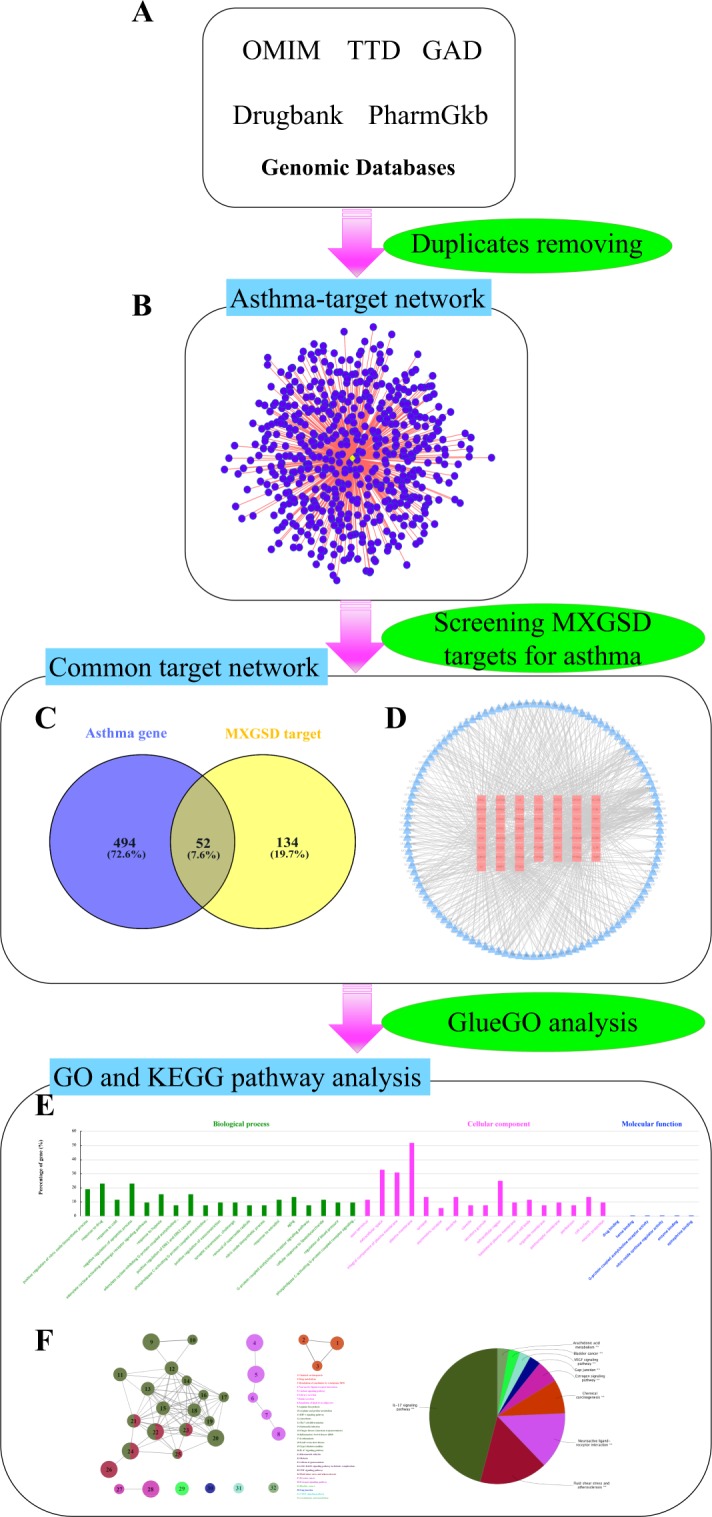
Asthma-related targets network. (**A**) Five disease gene target databases. (**B**) Asthma-target network, containing 542 nodes and 545 edges. (**C**) 52 common targets between asthma and MXGSD. (**D**) 52 common target network, containing 164 nodes and 764 edges. (**E**) GO and (**F**) KEGG pathway (bubble diagram on left, pie diagram on right) enrichment analysis of MXGSD on asthma.

Asthma is a polygenic genetic predisposing disease. Research on gene and gene-environment interaction is beneficial to revealing the pathogenesis of asthmatic diseases. In this study, we collected a total of 546 asthma-related targets (Fig. 2A) recorded in genomic databases of human diseases (Fig. 2B). Among these, MXGSD shared 52 common targets with asthma (Fig. 2C,D).

To gain insights into the pharmacological mechanisms of MXGSD on asthma, we performed GO and KEGG pathway analysis on the 52 common targets, and identified 19 BPs, 17 CCs, 6 MFs (Fig. 2E) and 32 KEGG pathways (Fig. 2F) of significance. The results were mainly enriched in airway inflammatory response, airway remodeling, and drug reaction, which were the same as the present pathologic mechanism of asthma. In addition, we also confirmed that MXGSD inhibits inflammation through the regulation of cholinergic, adrenergic, and other receptors with the involvement of nitric oxide biosynthesis, superoxide radicals removal, adrenergic receptor signaling pathway, and G-protein coupled acetylcholine receptor signaling pathway. Besides, ASM cell apoptosis and proliferation, arachidonic acid metabolism, interleukin (IL)-17 signaling pathway, and estrogen signaling pathway were also of great importance in clarifying the mechanisms of MXGSD on asthma. Asthmatics are increasingly being reported to also suffer from hypertension, hyperlipidemia, diabetes mellitus, heart disease and other comorbidities, which affects each condition and brings greater challenges to asthma treatment^64–66^. As shown by previous studies^67–69^, we also verified that MXSGD could treat asthma and regulate blood pressure, atherosclerosis, and diabetic complications simultaneously from the network pharmacology level. Asthma drugs can cause systemic changes but control asthma, but may exert a therapeutic or side effect on other diseases. Conversely, drugs regulating systemic changes, such as hormones and angiotensin converting enzyme inhibitors, can also affect asthma. Thus, we deduce that asthma may be a reaction of systemic neuro-immuno-inflammatory diseases in the respiratory system.

### MXGSD-asthma PPI network

**Figure 3:**
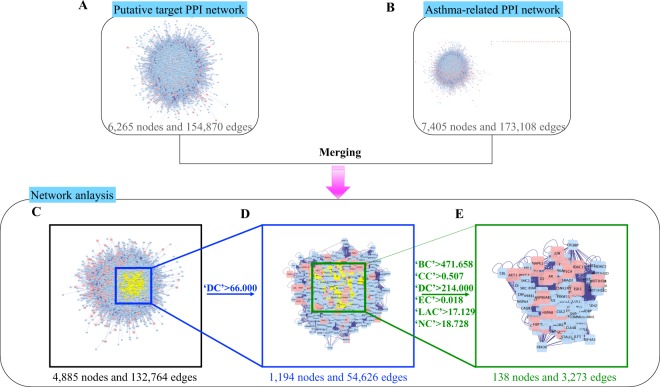
Construction of MXGSD-asthma PPI network. (**A**) MXGSD putative targets PPI network with 6265 nodes and 154 870 edges. (**B**) Asthma-related targets PPI network with 7405 nodes and 173 108 edges. (**C**) Interactive PPI network of MXGSD and asthma with 4885 nodes and 132 764 edges. (**D**) First central network evaluation with a core subset of 1194 nodes and 54 626 edges based on the median degree of 33. (**E**) Second central network evaluation with a core subset of 138 nodes and 3273 edges based on “‘BC’ > 471.658, ‘CC’ > 0.507, ‘DC’ > 214, ‘EC’ > 0.018, ‘LAC’ > 17.129, and ‘NC’ > 18.728”. Blue diamonds, pink diamonds, and yellow diamonds stand for other human proteins, component targets, and selected targets, respectively.

To dive deeper into the core pharmacological mechanisms of MXGSD in treating asthma, we first employed a topological method to evaluate the core network. PPI network is beneficial to understanding the role of various proteins in complex diseases, like asthma^70^. Therefore, we constructed MXGSD (Fig. 3A) and asthma-related targets (Fig. 3B) network with PPI databases, merged the two PPI network to obtain an interactive PPI network of MXGSD and asthma (Fig. 3C), before identifying the core PPI network according to the topology analysis screening criteria “DC ≥ 2 × median DC” (Fig. 3D). Eventually, we obtained 138 MXGSD core targets on asthma treatment, which satisfied the six screening criteria concurrently (Fig. 3E).

### Enrichment analysis of the 138 key targets

**Figure 4:**
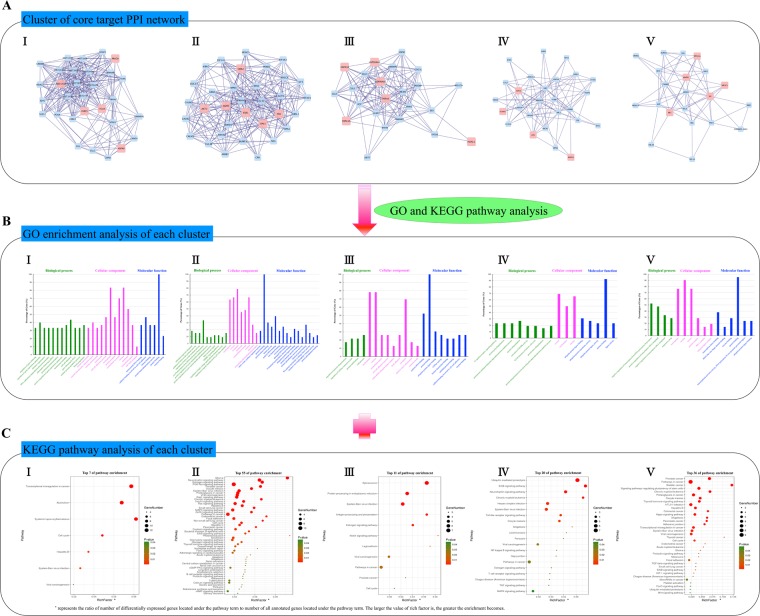
Enrichment analysis of the 138 core targets. (**A**) Clusters of the final central PPI network. Blue diamonds, pink diamonds, and yellow diamonds stand for other human proteins, component targets, and selected targets, respectively. (**B**) GO and (**C**) KEGG pathway enrichment analysis for every cluster.

To clarify the biological functions of the 138 key targets, we classified the final central PPI network into five clusters (Fig. 4A), and performed GO (Fig. 4B) and KEGG pathway enrichment analysis individually (Fig. 4C). Based on these GO terms data, we found that (i) gene expression, silence and replication; (ii) DNA/RNA damage repair and transcriptional regulation; (iii) protein classification, modification, synthesis and stability; (iv) signal transduction and cascade activation of inflammatory immune response; (v) DNA/RNA positive and negative regulation were enriched in their corresponding clusters, suggesting that MXGSD could treat asthma from a genetic perspective with multiple synergies.

Specifically, we found that many diseases such as systemic lupus erythematosus, hepatitis, leukemia, cancer, and other related biological molecules such as estrogen, prolactin, oxytocin, GnRH, aldosterone, renin, thyroid hormone, insulin, glucagon, gastric acid could also indirectly affect the development of asthma, providing strong evidence for our hypothesis that MXGSD could treat asthma through systemic neuro-immuno-inflammatory regulation from a genetic perspective.

In addition, based on the analysis of KEGG signaling pathway data, we believed that the key signaling pathways such as neurotrophin signaling pathway, estrogen signaling pathway, PI3K-Akt signaling pathway, and ErbB signaling pathway, the top 4 KEGG pathways in these clusters, might be the core pharmacological mechanism of MXGSD for asthma. All aforementioned analysis could shed light on a novel strategy for drug development on asthma.

## Discussion

**Figure 5:**
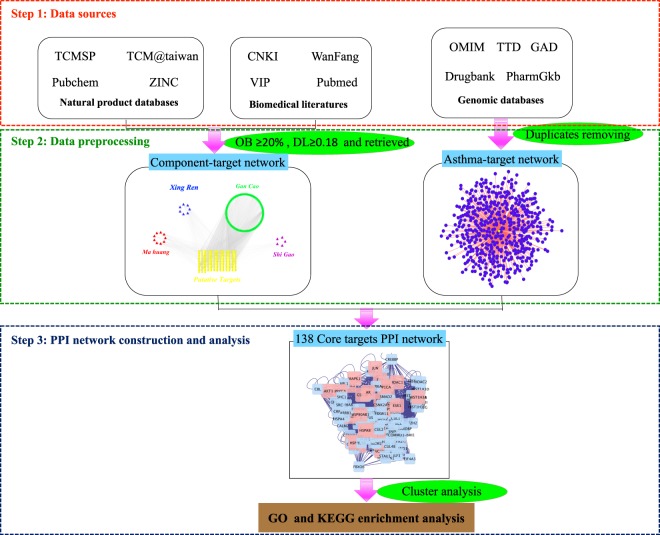
Diagram of the study design.

Asthma is a common complex respiratory disease with high morbidity and mortality. To date, pharmaceutical drugs are used mainly to control its related symptoms. Comparatively, TCM herbs not only control the symptoms, but may also resolve the underlying cause. MXGSD, through its thousand-year usage has demonstrated its practical effectiveness on asthma^71–73^, but the pharmacological mechanisms have not been uncovered systematically and comprehensively. In this study, we adopted network pharmacology to further explore the mechanisms of MXGSD on asthma. Three aspects aroused our attention: first, 52 common targets for asthma were enriched in pathological changes of airway inflammation and remodeling, as well as other diseases. Second, cluster analysis exhibited a refined detail of MXGSD on asthma compared to the common-target analysis. Thirdly, neurotrophin signaling pathway, estrogen signaling pathway, PI3K-Akt signaling pathway, and ErbB signaling pathway were considered as the key sections of MXGSD on asthma treatment from gene level. (The diagram of the study design is shown in Fig. 5).

Subsequently, we retrieved the four key signaling pathways of MXGSD associated with asthma treatment. Latest research shows that neurotrophic factors can be synthesized and released by a variety of immune and inflammatory cells, which mediate airway inflammation through increase of Th2 cells, eosinophils activation, induction of mast cell proliferation and differentiation, promotion of inflammatory mediators release, and immune response intensification^74^. Besides, neurotrophic factors stimulate airway epithelium sensory afferent nerve to synthesize neuropeptides^75^ and promote the differentiation of neural stem cells into cholinergic neurons^76–80^, which in return mediate airway inflammation, hyper-responsiveness, and remodeling. Combined with the regulation of MXGSD on other diseases as mentioned above, we deduce that asthma is a manifestation of the systemic neuro-immuno-inflammatory response in the respiratory system, which is also in line with the holistic comprehension of the physiological and pathological state of the human body in TCM.

Asthma is a polygenic genetic predisposing disease. The polymorphism of human genetic genes determines the body’s sensitivity and response to the diseases and drugs, as well as the diversity of clinical manifestations. This is similar to the concept of Z*heng* (pattern) in TCM – the summary of pathological properties at a certain stage in the development of disease, which is a functional state of the body responding to the pathogenic factors. Hereby, we speculate one of the theoretical bases of TCM is multi-gene in nature. In this study, we confirmed that MXGSD is effective for asthma from the levels of gene repair and expression to signal transduction and cascade activation in pathways. Meanwhile, some researchers emphasize on the relationships between polymorphisms of human genes and TCM pattern types/therapeutic effect from the perspective of clinical trials and experimental tests^81–83^. Therefore, multi-gene analysis may be a new research approach to verify the effectiveness of TCM herbs.

However, there are still some limitations. First, our collection of bioactive components and targets was not comprehensive. It can be improved by new detections using liquid chromatography, mass spectrometry, two-dimensional liquid chromatography or quadrupole-time-of-flight mass spectrometry. Second, our network pharmacology study lacked animal experiments and clinical trials to verify the hypothesis. In the future, we endeavor to verify the relationship between TCM pattern types and gene polymorphisms from animal experiments and clinical trials.

## Conclusions

In conclusion, we believe that asthma is a manifestation of the systemic neuro-immuno-inflammatory response in the respiratory system by network pharmacology. MXGSD not only relieves asthma, but also regulates the functional state of other systems in the human body from the gene level. Four signaling pathways of neurotrophin, estrogen, PI3K-Akt, and ErbB play vital roles in the treatment of asthma by MXGSD, providing a new direction for drug development on asthma.

## Electronic supplementary material

Dataset 1

Dataset 3

Dataset 4

Dataset 5

Dataset 6

Dataset 7

Dataset 8

Dataset 9

Dataset 10

Dataset 11

Dataset 12
