# Supplementary material for: Uncovering the mechanism of Maxing Ganshi Decoction on asthma from a systematic perspective: A network pharmacology study
Source: Sci Rep. 2018 Nov 26;8:17362. doi: 10.1038/s41598-018-35791-9 (PMC6255815; doi:10.1038/s41598-018-35791-9)
Supplement: Supplementary file 1 — Dataset 1 [file 41598_2018_35791_MOESM1_ESM.pdf]

Supplementary Information

Uncovering the mechanism of *Maxing Ganshi* Decoction on asthma from a systematic perspective: A network pharmacology study

Wenjie Song[1†], Shenglou Ni[2†], Yanling Fu[1*], Yun Wang[3]

[1]School of Traditional Chinese Medicine, Beijing University of Chinese Medicine, Beijing, 100029, China.

[2]Periodicals Publishing Center, Beijing University of Chinese Medicine, Beijing, 100029, China.

[3]School of Chinese Materia Medica, Beijing University of Chinese Medicine, Beijing, 100029, China.

[*]Corresponding Author: fuyanling@bucm.edu.cn.

[†]They contributed equally to this article.

1 Supplementary Tables

Supplementary Table S1 Components in MXGSD.

Supplementary Table S2 Known asthma-related targets.

Supplementary Table S3 Bioactive components in MXGSD.

Supplementary Table S4 Potential targets of bioactive components in MXGSD.

Supplementary Table S5 52 common targets between MXGSD and asthma.

Supplementary Table S6 GO and KEGG pathway analysis for the 52 common targets.

Supplementary Table S7 Network centrality analysis and evaluation.

Supplementary Table S8 Clusters of component-target and asthma-related target PPI interaction network.

Supplementary Table S9 GO analysis for each cluster.

Supplementary Table S10 KEGG pathway analysis for each cluster.
